# Emphysematous Cystitis: A Case Report and Literature Review

**DOI:** 10.7759/cureus.83351

**Published:** 2025-05-02

**Authors:** José Emiliano González Flores, Oscar Melin Herrera, Marco A Urbina Velázquez, Alfonso Sandoval Polito, Tania S Cortés Juárez, Carlos E Escobar Gómez

**Affiliations:** 1 Medicine, Instituto Tecnológico y de Estudios Superiores de Monterrey (ITESM) Campus Ciudad de México, Mexico City, MEX; 2 General Practice, Hospital General de Zona #36 Instituto Mexicano del Seguro Social (IMSS), Pachuca City, MEX; 3 Surgery, Hospital de Alta Especialidad de Veracruz, Veracruz, MEX; 4 Medicine, Hospital General de Zona #36 Instituto Mexicano del Seguro Social (IMSS), Pachuca City, MEX

**Keywords:** computed tomography diagnosis, diabetes mellitus complications., emphysematous cystitis, gram negative bacteria, urinary tract infections

## Abstract

Urinary tract infections (UTIs) are one of the most common infectious diseases worldwide, predominantly affecting women due to anatomical factors. Among UTIs, emphysematous cystitis (EC) is a rare but clinically significant entity characterized by gas presence in the bladder wall or lumen, often caused by gram-negative bacteria like *Escherichia coli* and *Klebsiella pneumoniae*. Diagnosis is challenging due to nonspecific symptoms such as dysuria, abdominal pain, and occasionally pneumaturia, with computed tomography being the diagnostic method of choice. We report the case of a 65-year-old male with diabetes and a significant surgical history, who was admitted for abdominal pain and nausea due to an intestinal obstruction. Incidental findings on computed tomography revealed EC, necessitating antimicrobial therapy adjustment. The patient was successfully managed conservatively with meropenem, glycemic control, and surgical follow-up for associated hernia complications. This case emphasizes the importance of the early recognition and multidisciplinary management of EC, particularly in high-risk patients like diabetics. Timely imaging and targeted antibiotic therapy are crucial for reducing complications and improving outcomes in such complex clinical scenarios.

## Introduction

Urinary tract infections (UTIs) constitute the second most common infectious disease worldwide, surpassed only by nosocomial respiratory infections in the United States and Europe [1]. UTIs can occur at any stage of life, with a notable increase in cases beginning in adolescence and the onset of sexual activity. They are more prevalent in women due to anatomical factors that facilitate microbial colonization [2].

Among UTIs, emphysematous cystitis (EC) is a rare but clinically significant entity characterized by the presence of gas within the lumen and bladder wall. It is secondary to severe infections caused by microorganisms, typically gram-negative bacteria such as* Escherichia coli* and *Klebsiella pneumoniae* [3]. Although infrequent, EC can be potentially fatal if not diagnosed and treated promptly, with a reported mortality rate ranging from 3% to 12%. However, among emphysematous conditions, such as emphysematous pyelonephritis (EP), mortality can reach up to 25% [4].

Diagnosing EC is challenging due to its atypical clinical presentation, which may include urinary symptoms such as dysuria, increased urinary frequency, suprapubic pain, and occasionally pneumaturia. Nonspecific symptoms depend on the underlying pathology and may include abdominal pain, nausea, and vomiting, which are among the most frequent [3]. Since uncomplicated UTIs generally do not require imaging studies for confirmation, detecting EC relies on radiological examinations, particularly computed tomography (CT), which reveals the presence of gas in the bladder wall or lumen [1].

Historically, the presence of air in the urinary tract was first reported in 1671 in a case of pneumaturia. Later, in the 19th century, Eisenlohr described cases of emphysematous bladder infections in autopsies, and in 1961, Bailey formally defined the condition as "emphysematous cystitis." This pathology predominantly affects women over 60 years of age with uncontrolled diabetes mellitus, occurring approximately twice as often in women as in men [5].

Early recognition of EC is essential, particularly in high-risk patients, such as diabetics and immunocompromised individuals, to reduce mortality and prevent severe complications. Treatment typically includes proper glycemic control, administration of broad-spectrum antibiotics, and bladder drainage via urethral catheterization. Timely intervention can lead to a favorable resolution of the infectious process and minimize the risk of complications [2].

## Case presentation

The case of a 65-year-old male patient with a significant surgical and metabolic history is presented. He was admitted to the emergency department with abdominal pain associated with nausea. During his evaluation, an intestinal obstruction secondary to a supraumbilical hernia was identified, a complication related to his previous surgical history. As an incidental finding, computed tomography revealed emphysematous cystitis, which required an adjustment in his antimicrobial management.

As relevant personal history, the patient had a diagnosis of type 2 diabetes mellitus, managed with glibenclamide (5 mg daily) and linagliptin (one tablet daily), as well as hypertension treated with losartan (50 mg daily). Regarding surgical history, he reported an appendectomy seven years ago, intestinal perforation treated with colostomy six years ago with subsequent bowel transit restoration, and the development of an incisional hernia and an enterocutaneous fistula, both managed conservatively for the past five years.

The current condition began with colicky abdominal pain accompanied by nausea, without vomiting, fever, diarrhea, or constipation. The patient self-medicated with pinaverium bromide without improvement. Upon arrival at the emergency department, his vital signs were as follows: blood pressure of 90/60 mmHg, heart rate of 80 beats per minute, respiratory rate of 20 per minute, temperature of 36°C, and oxygen saturation of 98%. Physical examination identified a distended abdomen due to a midline abdominal wall hernia extending from the epigastrium to the hypogastrium, irreducible, with tenderness on palpation in all quadrants. Additionally, an epigastric fistula was observed with scant, non-foul-smelling, greenish discharge and decreased peristalsis. The rest of the examination showed no relevant findings.

As initial management, intravenous crystalloid solutions, proton pump inhibitors, analgesics, prokinetics, a beta-lactam antibiotic, and a rapid-acting insulin regimen for hyperglycemia control were administered. A general surgery consultation was requested for further evaluation. During surgical assessment, the abdomen was found to be soft and depressible, with tenderness in the epigastrium and a 15 × 20 cm supraumbilical defect associated with an apparent loss of domain.

Initial laboratory studies showed hemoglobin of 15.0 g/dL, platelets of 317,000/µL, leukocytes of 17,450/µL, glucose of 348 mg/dL, urea of 45.7 mg/dL, blood urea nitrogen (BUN) of 21.36 mg/dL, creatinine of 1.59 mg/dL, chloride of 100.4 mEq/L, potassium of 4.9 mEq/L, sodium of 133.5 mEq/L, prothrombin time (PT) of 11.4 seconds, and partial thromboplastin time (PTT) of 28.6 seconds. A plain abdominal radiograph showed intestinal loop dilation, which, in the context of multiple previous surgeries and an enterocutaneous fistula, prompted an abdominal computed tomography scan and the placement of a nasogastric and urinary catheter. Table 1 shows the laboratory values obtained in the patient compared with the normal ranges.

**Table 1:** Laboratory values

Parameter	Patient (Observed Value)	Normal Range
Hemoglobin	15.0 g/dL	13.0 - 17.0 g/dL
Platelets	317,000/µL	150,000 - 450,000/µL
Leukocytes	17,450/µL	4,000 - 11,000/µL
Glucose	348 mg/dL	70 - 100 mg/dL (fasting)
Urea	45.7 mg/dL	7 - 20 mg/dL
Blood Urea Nitrogen	21.36 mg/dL	7 - 20 mg/dL
Creatinine	1.59 mg/dL	0.6 - 1.2 mg/dL
Chloride	100.4 mEq/L	98 - 106 mEq/L
Potassium	4.9 mEq/L	3.5 - 5.0 mEq/L
Sodium	133.5 mEq/L	136 - 145 mEq/L
Prothrombin Time	11.4 seconds	11 - 13.5 seconds
Partial Thromboplastin Time	28.6 seconds	25 - 35 seconds

**Figure 1:**
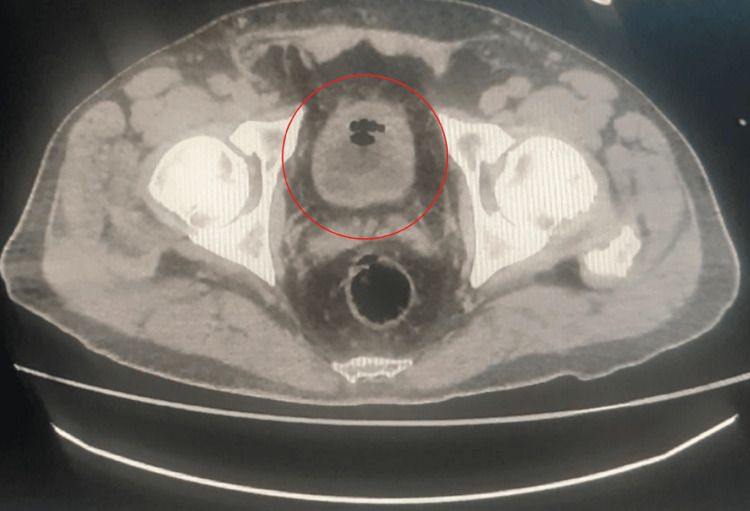
Axial projection of an abdominal CT scan Gas is observed within the lumen of the urinary bladder, consistent with emphysematous cystitis.

**Figure 2:**
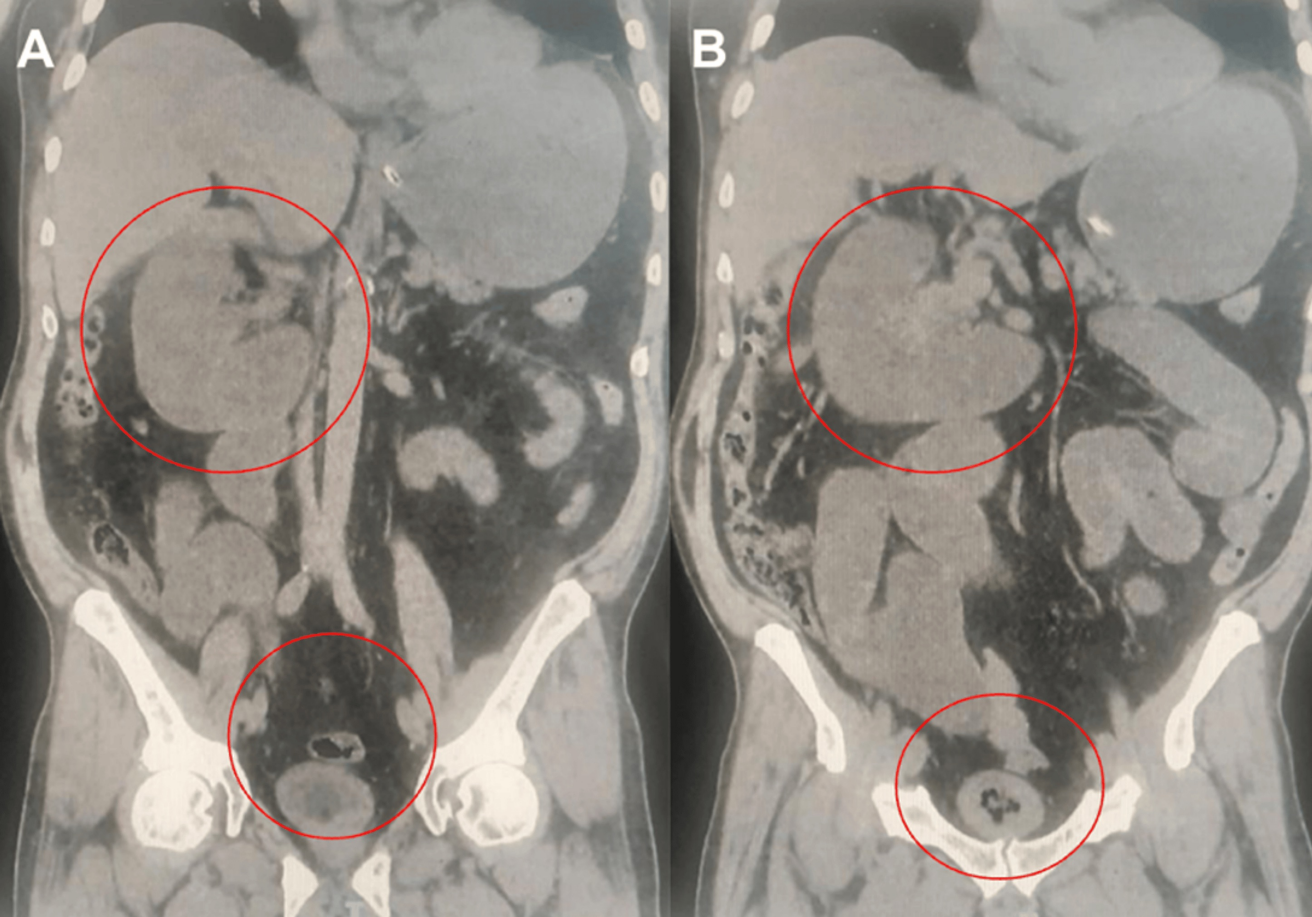
Coronal projection of abdominal CT scans (A) (B) Evidence of small bowel obstruction, inflammatory changes in the right kidney, and emphysematous cystitis

At 12 hours of hospital stay, the patient was reassessed, showing decreased pain, passage of gas, and unquantified urinary output via a Foley catheter. Physical examination revealed a mildly distended abdomen, a midline surgical scar, a fistula with minimal serous discharge, a midline hernia without signs of compromise, negative rebound tenderness, increased peristalsis in the left hemiabdomen, tympanic sounds, and no other relevant findings. The computed tomography scan showed small bowel obstruction likely secondary to the supraumbilical hernia, chronic inflammatory changes in both kidneys, and emphysematous cystitis (Figure 1) (Figure 2).

Conservative treatment included adjusting antimicrobial therapy to meropenem (1 g IV every 12 hours) to address emphysematous cystitis, along with measures for resolving the intestinal obstruction. Follow-up laboratory tests showed improvement, with hemoglobin at 13.2 g/dL, platelets at 212,000/µL, leukocytes at 6,160/µL, neutrophils at 4,190/µL, lymphocytes at 1,360/µL, glucose at 195 mg/dL, urea at 50 mg/dL, BUN at 23 mg/dL, creatinine at 0.97 mg/dL, chloride at 99 mEq/L, potassium at 4.1 mEq/L, and sodium at 134 mEq/L. The general urinalysis was unremarkable. After six days of hospitalization, the patient showed resolution of the intestinal obstruction and was discharged from the general surgery service with a referral to the complex hernia module for further management.

## Discussion

The case of a 65-year-old male patient with a significant surgical and metabolic history is presented. He was admitted with abdominal pain and nausea. The initial diagnosis was intestinal obstruction secondary to a complicated supraumbilical hernia. A computed tomography (CT) scan was performed to complement the diagnosis of intestinal obstruction, revealing chronic inflammatory changes in both kidneys and emphysematous cystitis, requiring an adjustment to antimicrobial therapy. Treatment included conservative management with fluid resuscitation, prokinetics, antibiotic therapy with meropenem 1 gram intravenously every 12 hours, glycemic control, and close monitoring, achieving resolution of the obstructive condition and improvement in inflammatory parameters. After six days of hospitalization, the patient was discharged with a follow-up for complex hernia management.

EC is a clinical entity characterized by the presence of gas within and around the bladder wall, produced by bacterial or fungal fermentation [6], and is most frequently associated with emphysematous pyelonephritis [7]. As mentioned in studies by Magallanes J et al. and Woodall C et al., the most common causative microorganism of EC is *Escherichia coli* (up to 75% of cases), followed by *Klebsiella pneumoniae* (20-30%). Less frequent cases may involve *Proteus mirabilis, Pseudomonas aeruginosa, Enterobacter aerogenes, Enterococcus spp., Staphylococcus aureus, Streptococcus spp., Nocardia, Clostridium perfringens, and Candida albicans* [3,7].

According to Amano N et al., emphysematous cystitis presents with multiple clinical findings that range from abdominal pain to macroscopic hematuria, urinary retention, and pneumaturia [5]. However, these symptoms are nonspecific and generally mild, and no specific clinical evidence has been reported to suggest the presence of this entity. Up to 7% of patients may remain asymptomatic.

The main risk factors associated with EC include diabetes, neurogenic bladder, immunosuppression, and chronic infection [5]. Specifically, older women with uncontrolled diabetes have a higher risk of developing emphysematous cystitis. The condition shows a 2:1 female-to-male predominance, and two-thirds of reported cases occur in diabetic patients [8]. Diabetes mellitus, particularly in poorly controlled patients, is a key risk factor for EC due to increased glucose in the interstitial fluid, which promotes bacterial fermentation and gas production. However, in non-diabetic patients, microorganisms may utilize urinary lactate or tissue proteins as a substrate for gas generation [9].

CT is the imaging modality of choice for diagnosing emphysematous cystitis and other conditions involving intravesical gas, as it has a high sensitivity for detecting intramural or intraluminal gas. The characteristic CT finding of EC is the presence of air bubbles in the bladder wall or lumen in the absence of recent instrumentation. Another imaging modality that can be useful for detecting EC findings is magnetic resonance imaging (MRI), as it can reveal inflammatory changes consistent with concurrent cystitis or the presence of fistulas involving the bladder [1]. As in the presented case, EC is often an incidental finding in imaging studies conducted for the diagnostic evaluation of other conditions. In this case, the patient presented with an intestinal obstruction, and a CT scan revealed emphysematous cystitis incidentally [10].

Although EC is often an incidental finding, its timely diagnosis is crucial, as delays can result in the spread of infection to the renal parenchyma or ureters, increasing the risk of severe complications such as emphysematous pyelonephritis, sepsis, necrosis, and subcutaneous emphysema [11]. Given the potential for multiple coexisting conditions in a single patient, it is essential to consider all differential diagnoses when evaluating the causes and prognosis of EC. However, in most cases, patients recover after appropriate antibiotic therapy. EC can progress to severe systemic complications, including multi-organ failure affecting the kidneys, liver, and/or lungs [12], contributing to an approximate mortality rate of 7-7.4% when considered as an individual entity [10]. When associated with emphysematous lesions in other organs, such as emphysematous pyelonephritis, mortality ranges from 15.4% to 21%, according to various studies [4,13].

Since EC is bacterial in most cases, treatment is based on broad-spectrum antibiotics such as quinolones, penicillins combined with beta-lactamase inhibitors, carbapenems, or third-generation cephalosporins. In the presented case, the adjustment of antimicrobial therapy to meropenem 1 gram intravenously every 12 hours was essential due to the incidental finding of EC on CT, aligning with previous reports suggesting that conservative treatment is generally sufficient in the absence of major complications. If imaging studies reveal severe involvement due to extravesical abscess formation, surgical management should be considered [2]. Table 2 provides a comparative analysis of reported cases from the referenced sources in this article.

**Table 2:** Studies reporting emphysematous cystitis CT: computed tomography, AC: amoxicillin with clavulanic acid, GCS: Glasgow Coma Scale

Study	Initial Symptoms	Diagnosis	Isolated Pathogen (Complication)	Treatment	Resolution	Level of Evidence
Li, et al (2018) [4]	Nausea, Vomiting, and Abdominal Pain	CT Scan	Blood Culture: Gram-Negative bacilli; Urine Culture: Candida albicans	Meropenem and percutaneous drainage of the right kidney (due to emphysematous pyelonephritis)	Infection Remission	V
Chávez, et al (2020) [6]	Weakness, Fever, Headache, and Vomiting	CT Scan	Escherichia coli	Ceftriaxone and Meropenem	Infection Remission	V
Woodall, et al (2014) [7]	Nausea and Vomiting Resistant to Medical Treatment for 1 Day	CT Scan	Urinalysis Without Evidence of Infection	Fosfomycin	Infection Remission	V
De Coninck, et al (2015) [8]	Case 1: Pyuria and Hematuria Case 2: Acute Confusional Syndrome and Abdominal Pain	Case 1: CT Scan; Case 2: CT Scan	Case 1: Escherichia coli; Case 2: Escherichia coli	Case 1: Temocillin; Case 2: Ciprofloxacin	Case 1: Infection Remission in 10 Days; Case 2: Infection Remission in 7 Days	V
Irusta, et al (2014) [9]	Abdominal Pain, Increased Urinary Frequency, Dysuria, and Macroscopic Hematuria	CT Scan	No Urinalysis Performed	AC	Infection Remission	V
Yousra, et al (2024) [11]	Asymptomatic	CT Scan	Escherichia coli	Placement of urinary catheter and management with ceftriaxone followed by ciprofloxacin	Infection Remission	V
Kawaguchi, et al (2024) [10]	Hypotension, ECG 10, Body Temperature of 38°C, and Macroscopic Hematuria	CT Scan	Klebsiella pneumoniae	Meropenem and placement of a JJ catheter with periodic replacement	Sequelae of Prominent Vesicoureteral Reflux Without Signs of Pyuria. Deceased 7 Months After Initial Diagnosis Due to Dysphagia.	V
Puerta, et al (2017) [12]	Epigastralgia Associated with Nausea and Pneumaturia	CT Scan	Escherichia coli	Piperacillin with tazobactam	Developed Multiorgan Failure During Hospitalization, with Infection Remission	V
Wang, et al (2018) [13]	Fever (Unspecified Temperature) with One-Week Evolution	CT Scan	Escherichia coli	Placement of urinary catheter and management with levofloxacin	Infection Remission	V
Babinger, et al (2023) [14]	Abdominal Pain, Diarrhea, and Acute Confusional Syndrome	CT Scan	Escherichia coli	Ceftriaxone	Infection Remission	V
Mizuno, et al (2016) [15]	Acute Confusional Syndrome and Distended Lower Abdomen	CT Scan	Escherichia coli	Ceftriaxone and cephalexin	Infection Remission	V

## Conclusions

The successful management of this case highlights the importance of considering incidental pathologies, such as emphysematous cystitis, within the patient's overall clinical context. The presence of risk factors, including diabetes mellitus and prior surgical history, underscores the need for rigorous monitoring and an individualized approach to optimize outcomes. Computed tomography played a crucial role not only in confirming the diagnosis of EC but also in assessing the extent of associated complications, guiding appropriate antimicrobial management.

This case emphasizes the significance of a comprehensive approach and interdisciplinary collaboration in managing patients with coexisting metabolic and surgical conditions. The implementation of evidence-based therapeutic strategies, such as the use of carbapenems, was essential for effective infection control and patient recovery, demonstrating the positive impact of a multidisciplinary intervention.
